# Anesthetic management of a patient with achalasia, a disease with a considerable risk for aspiration under anesthesia

**DOI:** 10.1186/s40981-023-00650-8

**Published:** 2023-09-07

**Authors:** Keiko Haraguchi-Suzuki, Chizu Aso, Masashi Nomura, Shigeru Saito

**Affiliations:** 1 Intensive Care Unit, Gunma University Hospital, 3-39-15 Showa, Maebashi, Gunma 371-8511 Japan; 2 Department of Anesthesiology, Gunma University Hospital, 3-39-15 Showa, Maebashi, Gunma 371-8511 Japan; 3 Department of Urology, Gunma University Hospital, 3-39-15 Showa, Maebashi, Gunma 371-8511 Japan

**Keywords:** Achalasia, Rapid sequence induction, Regurgitation, Aspiration

## Abstract

**Background:**

Achalasia is a rare condition characterized by dysfunction of esophageal motility and impaired relaxation of the lower esophageal sphincter. Anesthetic management of these patients is challenging due to the elevated risk of regurgitation and aspiration.

**Case presentation:**

A 53-year-old man diagnosed with achalasia was scheduled for renal cancer surgery before esophageal myotomy. Since his severe dysphagia suggested the possibility of vomiting and aspiration under anesthesia, a stomach tube was inserted before induction of general anesthesia. After preoxygenation, rapid sequence induction was performed and an antiemetic was administered to prevent postoperative vomiting. Although anesthetic management was uneventful, the inserted stomach tube coiled up in the dilated esophagus and substantial residue was aspirated via the tube even after a prolonged fasting period.

**Conclusion:**

Anesthesiologists should be familiar with achalasia even though it is an uncommon disease, since affected patients are at risk of regurgitation and aspiration under anesthesia.

## Background

Achalasia is a motility disorder of the esophagus, characterized by aperistalsis and impaired relaxation of the lower esophageal sphincter (LES). The etiology of achalasia is considered to involve selective loss of neurons in the myenteric plexus in the distal esophagus and lower esophageal sphincter. This leads to lack of release of nitric oxide and vasoactive intestinal peptide, leading to inadequate relaxation of the esophageal smooth muscle and lower esophageal sphincter [1]. Since achalasia patients experience dysphagia and occasional vomiting, some patients develop progressive weight loss. Although achalasia is a rare disease with a prevalence of about 9–10 in 100,000 people, being even more rare in children and adolescents except for those with genetic disorders such as Down syndrome, these patients are at risk of regurgitation under anesthesia and aspiration pneumonia during the perioperative period [2–4].

Aspiration is an emergency situation under anesthesia, which can complicate airway management, such as assisted ventilation and intubation. Additionally, aspiration worsens the patient’s prognosis because it causes both chemical and bacterial pneumonia due to aspiration of gastric acid and anaerobic bacteria from the oral cavity [5]. Thus, preventing aspiration is an important part of anesthesia management. Achalasia patients are prone to developing regurgitation during surgery due to an increase in esophageal pressure in the supine position and relaxation of the gastroesophageal junction after administration of anesthetics. Here, we report the anesthetic management of a patient with severe achalasia symptoms who was scheduled to undergo renal cancer surgery.

## Case presentation

**Fig. 1:**
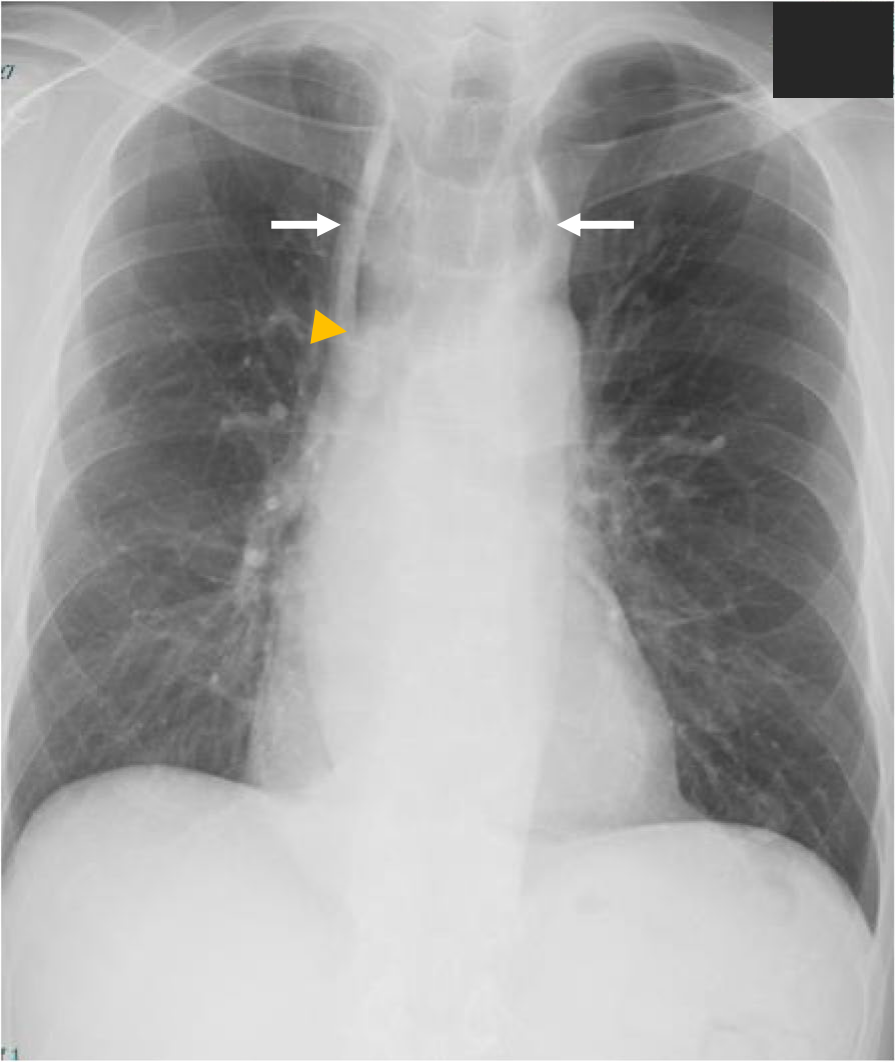
Chest X-ray demonstrated enlargement of the mediastinum (white arrows) and presence of air-fluid levels in the thoracic esophagus (yellow arrowhead)

**Fig. 2:**
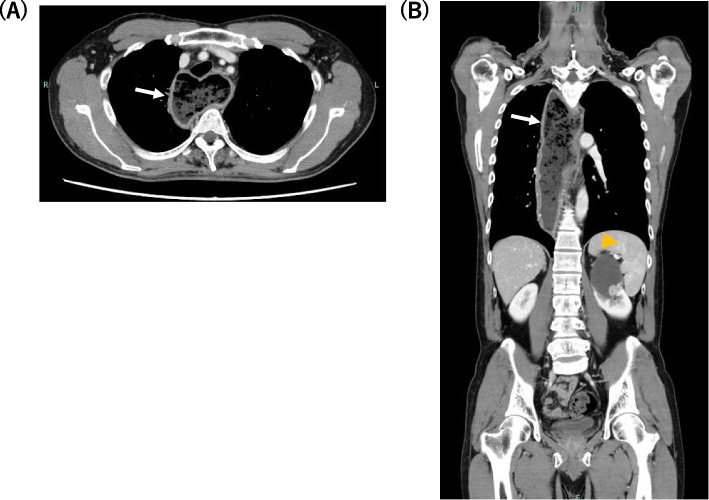
**A** Axial computed tomography (CT) image showing the enlarged esophagus filled with residue (arrow). **B** Coronal CT image showing dilation of the upper part and narrowing of the lower part of the esophagus, presenting a bird beak shape (white arrow). The left renal tumor is also seen (yellow arrowhead)

A 53-year-old man, 168 cm tall, weighing 60.4 kg (body mass index 21.4 kg/m^2^), and suffering from difficulty in swallowing since his mid-teens, presented for treatment of the dysphagia. His symptoms worsened in winter and had progressively worsened over time. His medical history included autoimmune hepatitis, and he had no family history of gastrointestinal tract diseases. Chest X-ray showed mediastinal enlargement and presence of air-fluid levels in the thoracic esophagus (Fig. 1). He underwent upper gastrointestinal endoscopy along with biopsy, which showed a dilated esophagus with food residue and constriction of the esophagogastric junction. He was diagnosed with achalasia type 2 according to the Chicago classification, indicating that his esophagus did not have effective movement [6]. He was scheduled for endoscopic myotomy of the esophagus under general anesthesia. Preoperative computed tomography imaging revealed the shape of the esophagus, with an enlarged upper part and narrowed lower part containing much residue (Fig. 2A). Additionally, he was found to have a left renal tumor and was scheduled for renal cancer surgery before esophageal myotomy (Fig. 2B).

**Fig. 3:**
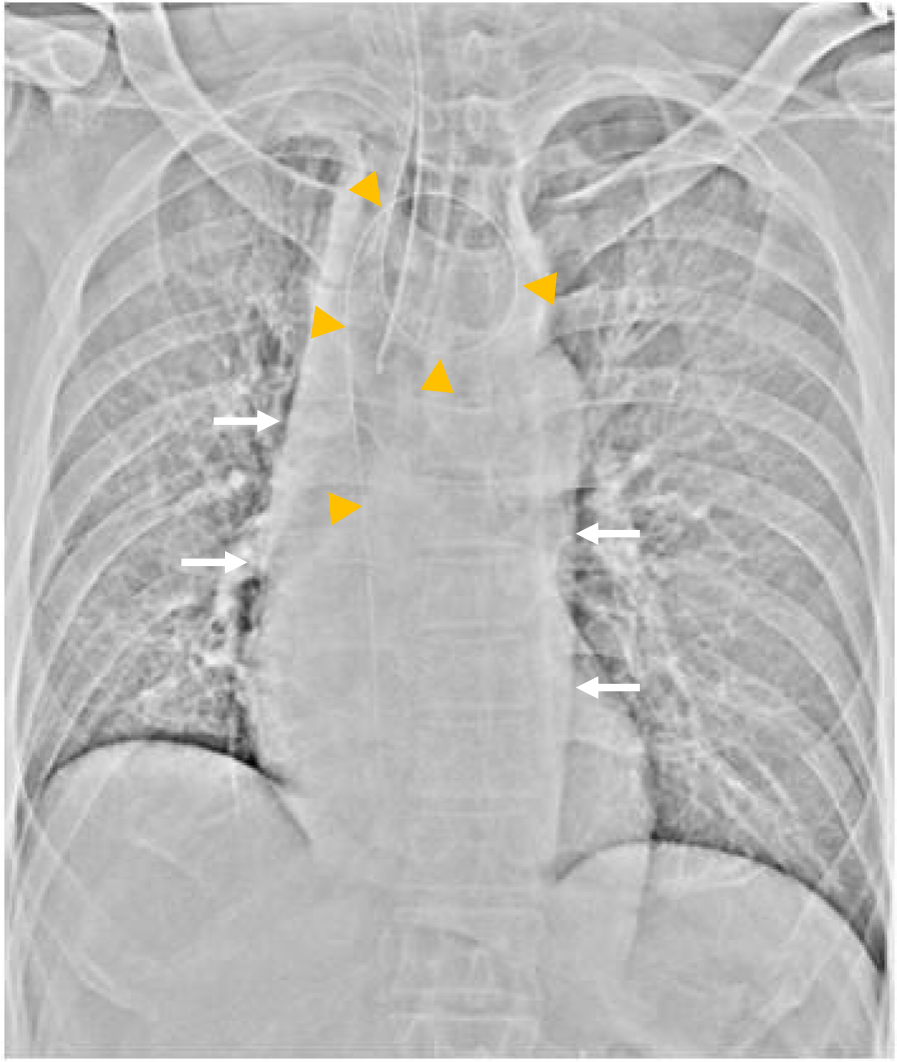
Chest X-ray image demonstrating enlargement of the mediastinum (white arrows). The stomach tube had formed a loop in the dilated esophagus (yellow arrowheads)

When he entered the operation room for the renal surgery, his vital signs were a heart rate of 74 beats/min, blood pressure of 132/92 mmHg, and SpO_2_ of 98%. Before induction of anesthesia, a 14-Fr stomach tube was inserted to a depth of 55 cm from his left nostril. Even though he had been fasting for 15 h, approximately 300 ml of residue was aspirated via the tube. Subsequently, after preoxygenation with 100% oxygen for 5 min, rapid sequence induction (RSI) was performed in the supine position with 110 mg of propofol, 60 mg of rocuronium bromide, 0.2 μg/kg/min of remifentanil, and 0.1 mg of fentanyl, using video laryngoscopy. Although the cricoid pressure (CP) maneuver was not applied along with RSI, intubation was successfully performed without regurgitation. Laparoscopic nephrectomy was performed in the right lateral recumbent position. Anesthesia was maintained with air, oxygen, desflurane, and remifentanil. To prevent regurgitation in case of bucking and elevated esophageal pressure due to pneumoperitoneum and the intraoperative position, rocuronium bromide was intermittently administered under neuromuscular monitoring using acceleromyography. The train of four (TOF) count was used to monitor the state of muscle relaxation. The total blood loss and urinary volume were 2 ml and 250 ml, respectively. The volume of infusion was 2298 ml during anesthesia. At the end of the operation, chest X-ray showed that the stomach tube had formed a loop in the dilated esophagus and did not reach the stomach (Fig. 3). After turning the patient over to the supine position and before extubation, the contents of the esophagus were again aspirated via the tube, although only minimal residue was obtained. The 5-HT_3_ receptor antagonist, ondansetron, was administered to prevent postoperative nausea and vomiting (PONV). Since the TOF count was 1 at the end of the surgery, 200 mg (3.3 mg/kg) of sugammadex was administered, resulting in recovery of the TOF count to 4. Finally, his trachea was extubated after recovery of spontaneous respiration without regurgitation at the supine position. The operation time was 4 h 33 min, and anesthesia time was 5 h 36 min.

At his exit from the operation room, the patient’s vital signs indicated a heart rate of 63 beats/min, blood pressure of 83/50 mmHg, and SpO_2_ of 99%. He was postoperatively observed in the intensive care unit (ICU). His postoperative pain was well controlled with 1 ml/h of patient-controlled analgesia consisting of 25 μg/ml fentanyl and 125 μg/ml droperidol, without PONV. He was discharged from the ICU on postoperative day 1, and his subsequent postoperative course was uneventful.

## Discussion

Achalasia is a motility disorder of the esophagus with dysfunction of relaxation of the LES. The primary concern in achalasia patients under general anesthesia is aspiration caused by regurgitation. To prevent regurgitation, RSI with the application of cricoid pressure (CP) is occasionally performed. However, the technique of CP is difficult since the appropriate pressure to be applied on the cricoid cartilage might differ between patients [7]. It is also difficult to understand the positional relationship between the cricoid cartilage and dilated esophagus in achalasia patients. Thus, we did not apply CP as part of the RSI maneuver. A stomach tube was instead inserted, and the patient was adequately preoxygenated before anesthesia induction. Suction of the gastroesophageal contents resulted in aspiration of a surprisingly large volume of residue via the tube despite 15 h of fasting. This suggests that achalasia patients should be prescribed a low residue diet before fasting, along with a longer fasting period than usual. Furthermore, although the RSI maneuver is usually applied for achalasia patients during general anesthesia, there is no evidence of a reduced incidence of aspiration with the application RSI [8–10]. We think that preoperative aspiration of residue via a stomach tube in the dilated esophagus is a highly effective way in preventing regurgitation and aspiration pneumonia before induction for achalasia patients.

The esophagus in achalasia patients presents a “bird’s beak” shape due to the dilated esophagus resulting from insufficient relaxation of the LES (Fig. 1) [11]. As shown in Fig. 3, the inserted stomach tube formed a loop in the dilated esophagus and did not reach the stomach in our patient. Since insertion of the stomach tube in patients with achalasia is difficult due to the shape of the esophagus, endoscope-guided insertion of a large diameter tube, if possible, before the scheduled operation is ideal. Furthermore, adoption of the reverse Trendelenburg position might be an effective way of decreasing aspiration in cases at risk of regurgitation during induction and extubation.

Previous case reports have described unpredicted vomiting and aspiration during performance of assisted ventilation at anesthesia induction in patients with undiagnosed achalasia [12, 13]. Routine preoperative chest X-ray examination showing enlargement of the mediastinum and presence of air-fluid levels in the thoracic esophagus might be indicative of achalasia [14]. Additionally, achalasia patients might have symptoms such as dysphagia, nocturnal coughing, and vomiting. Some patients might also have peculiar habits, such as consuming large volumes of water at meal times and vomiting every night before sleep [12, 13]. Asking questions related to such symptoms and habits during the perioperative examination are helpful in diagnosing achalasia. A previous significant report described an adolescent girl with achalasia who was misdiagnosed with asthma due to the symptoms of nocturnal coughing and dyspnea [15]. This suggests that achalasia should be included in the differential diagnosis of patients with asthma who present mainly with nocturnal symptoms and are refractory to treatment. We think that preoperative respiratory function evaluation might also be useful in distinguishing achalasia from the obstructive ventilatory impairment of asthma. Preoperative inquiry, in addition to usual examination, including chest X-ray and respiratory function tests, is useful for determining undiagnosed achalasia and preventing unexpected aspiration.

Our patient was subsequently scheduled for Heller myotomy of the LES 4 months after the kidney surgery under general anesthesia. We think that the knowledge and experience obtained from this case provides useful information for the safe anesthetic management of patients with achalasia.

## Conclusion

Achalasia is an important disease for anesthesiologists since its anesthetic management is challenging because of the risk of aspiration. Prevention of aspiration using a stomach tube to remove the residue accumulated in the dilated esophagus and prevention of postoperative vomiting are important aspects of the anesthetic management of patients with achalasia.

## Data Availability

Data sharing is not applicable to this article as no datasets were generated or analyzed during the current study.

## Abbreviations

LES: Lower esophageal sphincter
SpO_2_: Oxygen saturation in the peripheral artery
RSI: Rapid sequence induction
CP: Cricoid pressure
TOF: Train of four
ICU: Intensive care unit
5-HT_3_: 5-Hydroxytryptamine 3
